# Manipulation of Ferroic Orders via Continuous Biaxial Strain Engineering in Multiferroic Bismuth Ferrite

**DOI:** 10.1002/advs.202417165

**Published:** 2025-03-26

**Authors:** Jiesu Wang, Shuai Xu, Sebastian Meyer, Shiyao Wu, Subhadeep Bandyopadhyay, Xu He, Qiyuan Miao, Sisi Huang, Pengzhan Li, Kun Zhao, Er‐Jia Guo, Chen Ge, Bertrand Dupé, Philippe Ghosez, Kai Chang, Kuijuan Jin

**Affiliations:** ^1^ Beijing Academy of Quantum Information Sciences Beijing 100193 China; ^2^ Beijing National Laboratory for Condensed Matter Physics Institute of Physics Chinese Academy of Sciences Beijing 100190 China; ^3^ University of Chinese Academy of Sciences Beijing 100049 China; ^4^ TOM research group Q‐MAT research unit Université de Liège Liège B‐4000 Belgium; ^5^ Theoretical Materials Physics Q‐MAT research unit Université de Liège Liège B‐4000 Belgium; ^6^ Institute of Ultrafast Optical Physics Department of Applied Physics and MIIT Key Laboratory of Semiconductor Microstructure and Quantum Sensing Nanjing University of Science and Technology Nanjing 210094 China

**Keywords:** antiferromagnetic, bismuth ferrite, first‐principles calculations, multiferroics, second harmonic generation, strain manipulation

## Abstract

Continuous strain engineering of multiferroics not only enhances understanding of their properties but also guides the optimization of their performances for use in electronic, optical, and magnetic devices. However, due to technical challenges in real‐time monitoring of the ferroic orders, the precise evolution process remains unclear. Here, the evolution of the ferroelectric (FE) and antiferromagnetic (AFM) orders are revealed in multiferroic freestanding BiFeO_3_ films under sequential and anisotropic biaxial strain, using rotational anisotropy second harmonic generation (RA‐SHG) technology and first‐principles calculations. The change and recovery of RA‐SHG patterns illustrate the reversible control of the in‐plane FE polarization in the films by sequential strain application. The in‐plane FE direction can be manipulated within ≈4° by strain along the (100) and (010) directions, while the AFM order is more significantly affected, with ≈8° rotation in RA‐SHG patterns. This research unveils the appearance of new SHG peaks in freestanding BFO films under strain and shows that they evolve independently of FE‐induced SHG linked to lattice changes, suggesting a spin structure‐related variation. This work paves a new way for studying of strain‐manipulated 2D multiferroics and highlights the promise of freestanding perovskite films as low‐dimensional multifunctional devices.

## Graphical abstract

This study explores the evolution of ferroelectric (FE) and antiferromagnetic (AFM) orders in freestanding BiFeO_3_ films under anisotropic strain using rotational anisotropy second harmonic generation (RA‐SHG) and first‐principles calculations. The research reveals reversible control of in‐plane FE polarization and significant AFM order rotation, discovering new SHG peaks linked to spin structure variations. These findings highlight the potential of strain‐engineered perovskite films for low‐dimensional multifunctional devices.

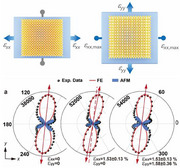

## 1 Introduction

Revisited more intensively for two decades, Bismuth Ferrite (BiFeO_3_, BFO) manifests noncollinear G‐type antiferromagnetic (AFM) order coupled with ferroelectric (FE) order at room temperature.^[^ ^1^, ^2^, ^3^, ^4^ ^]^ As such, it has emerged as a central focus in materials research, being one of the rare room‐temperature single‐phase magnetoelectric multiferroic materials.^[^ ^5^, ^6^, ^7^, ^8^ ^]^ Its robust ferroelectric properties, promising magnetoelectric coupling, and exceptional optical behavior not only make it a prototypical compound for fundamental research but also position it as a very promising candidate for potential applications.^[^ ^9^, ^10^ ^]^ The true significance of these attributes also lies in their adaptability through controlled modifications. While the application of magnetic and electric fields,^[^ ^6^, ^11^, ^12^, ^13^ ^]^ along with chemical doping,^[^ ^13^, ^14^, ^15^ ^]^ have proven their effectiveness in modulating the properties of BFO, strain engineering presents an additional compelling avenue for refining the remarkable functions of perovskite oxides and exploring their phase diagrams.^[^ ^13^, ^16^, ^17^, ^18^, ^19^ ^]^ However, the challenge persists in applying in situ continuous and adjustable strain to BFO, rendering the physical mechanism at play in such scenarios an unresolved issue.

A groundbreaking approach involves the crafting of flexible and freestanding BFO films through sacrificial layer techniques.^[^ ^20^, ^21^ ^]^ These films can be seamlessly transferred onto flexible organic substrates, such as polydimethylsiloxane (PDMS), enabling convenient and continuous tensile strain control. This method effectively addresses the limitations of substrate‐based approaches, which often grapple with discrete strain. Consequently, the exploration and application of BFO enter a new realm of possibilities. Recently, research on the strain manipulated freestanding BFO film has been reported, which was mainly focused on the uniaxial strain effects and on its flexoelectric effect,^[^ ^22^ ^]^ thermal transportation,^[^ ^23^ ^]^ electrical polarization,^[^ ^24^ ^]^ optical anisotropy,^[^ ^17^ ^]^ magnetic spin order,^[^ ^25^ ^]^ etc.

Limited by the typically large size of the stretching devices designed for applying continuous (biaxial) strain to these freestanding oxide films, their integration into characterization systems such as ferroelectric testers, piezo‐force microscopes, and scanning tunneling microscopes is challenging. Consequently, the characterization of the ferroelectric properties under continuous (biaxial) strain control is particularly difficult. Moreover, the anti‐parallel spin arrangement in antiferromagnets results in zero stray fields, making it hard to study using traditional near‐field imaging techniques.^[^ ^7^, ^25^ ^]^ Therefore, capturing and analyzing the evolution of FE order, AFM order, and magnetoelectric coupling in freestanding BFO films under strain modulation remains elusive. However, the polarization of BFO does not only break the spatial inversion symmetry of crystal structure, but also the spatial inversion of spin lattice structure, offering potential solutions. Hence, the use of optical second harmonic generation (SHG) technology, highly sensitive to the spatial inversion symmetry, allows us to monitor and characterize the evolution of the two types of ferroic orders under continuous strain modulation in‐situ and in‐time. Furthermore, a substantial body of prior work suggests that optical SHG techniques have successfully characterized the magnetoelectric coupling effects in multiferroic oxides or 2D materials.^[^ ^7^, ^25^, ^26^ ^]^

In this work, we utilize a custom‐designed in‐situ stretching optical instrument, complemented by optical SHG technology, to explore both FE and AFM orders of freestanding BFO (001) films under continuous and anisotropic biaxial strain. The strain is applied sequentially, first along the (100) direction and subsequently in the (010) direction. It is found that the shape and intensity of the rotational anisotropy second harmonic generation (RA‐SHG) patterns, showing the second harmonic signal as a function of the rotation angle of the polarization of the incident and transmitted light and the crystal axes, undergo alterations under the application of strain along the *x*‐axis. These alterations are reversible when the vertical strain along the *y*‐axis is engaged. Theoretical fittings of RA‐SHG patterns show that the FE and AFM orders in ≈5‐nm‐thick BFO films were respectively regulated by ≈4° and 8° in‐plane under the uniaxial tensile strain of ≈1.5%, which were further supported by first‐principles calculations. Meanwhile, the strain results in a ≈58.7% enhancement of SHG intensity in the multiferroic films, which is so far the largest enhancement for strain‐induced SHG in 2D or freestanding materials. We also demonstrate that the photoelastic coefficient of freestanding BFO films is strain dependent. This work provides new insights on the strain‐manipulated multiferroic orders and provides a promising approach to visualize their evolutions, in‐situ and in real‐time. These findings unlock possibilities for employing freestanding ferroic oxide films in low‐dimensional nonlinear optical applications and devices.

## 2 Results and Discussion

### 2.1 Properties of freestanding BiFeO_3_ films

**Figure 1:**
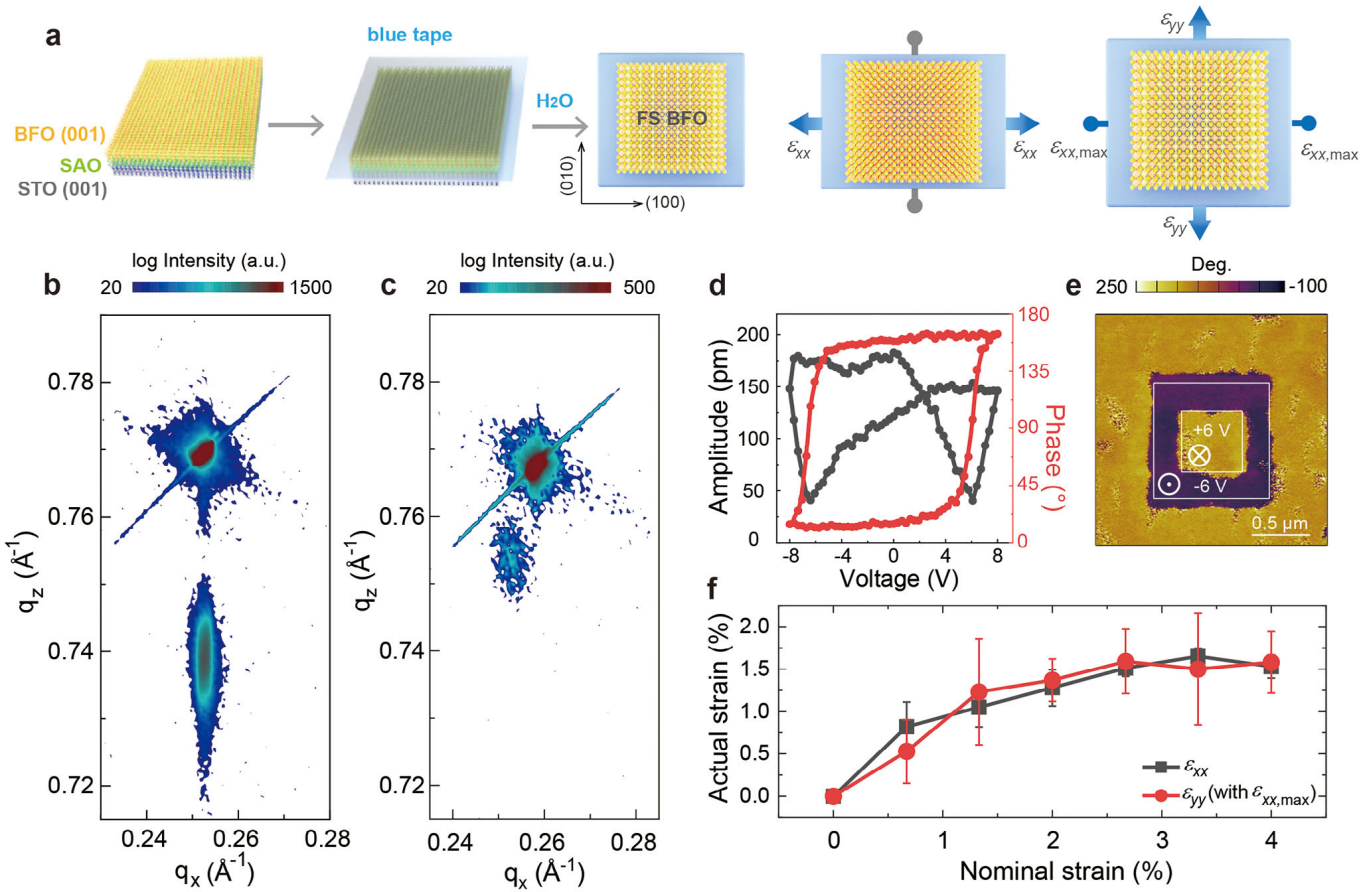
Preparation, surface, and ferroelectric properties of freestanding BFO films. a) Schematics of fabrication and anisotropic biaxial strain applied process of freestanding BFO films. b,c) RSM results of ≈20‐nm‐thick epitaxial and freestanding BFO films around (013) peak, respectively. d,e) Local PFM phase (red) as well as amplitude (black) hysteresis loops and out‐of‐plane PFM phase images of the ≈20‐nm‐thick freestanding BFO films, respectively. f) Relationship between actual strain on BFO films and nominal strain on blue tape.

High‐quality epitaxial BFO/Sr_4_Al_2_O_7_(SAO)/SrTiO_3_ (STO) (001) heterostructures with different thicknesses of BFO layers are synthesized by laser‐molecular beam epitaxial (laser‐MBE) method, and then freestanding BFO films are lifted off the STO substrates by dissolving the SAO sacrificial layer in de‐ionized water,^[^ ^21^, ^22^ ^]^ as schematically shown in **Figure** **1**a. The thicknesses of BFO are 4.2, 20.5, and 42.4 nm, respectively, defined by X‐ray reflectivity (XRR) as shown in Figure S1a (Supporting Information). X‐ray diffraction (XRD) *θ*–2*θ* curves (Figure S1b, Supporting Information) and reciprocal space mapping (RSM) results (Figure 1b and Figure S2, Supporting Information) demonstrate that there is no secondary phase or twinning structure, indicating the BFO layers are totally constrained by the substrates. For the freestanding ones, there is no splitting in the (013) diffraction spot (Figure 1c), demonstrating the consistence in freestanding BFO films despite the constraint relief and the presence of surface wrinkles.^[^ ^8^, ^22^ ^]^ No diffraction spots around (013) direction can be observed for the ≈5‐nm‐thick freestanding BFO film, perhaps due to the thickness—the X‐ray diffraction intensity of the freestanding films is significantly reduced compared to the epitaxial films, and that of the ≈5 nm films is already weak in its epitaxial form. The reversible ferroelectricity of the freestanding BFO films is proven by local piezoelectric force microscopy (PFM) hysteresis loops (Figure 1d) with almost 180° phase flips and the PFM images (Figure 1e) with sharp contrast between up and down ferroelectric domains written by PFM tip. To do the PFM experiments, the freestanding BFO films were transferred on conducting Nb‐doped STO substrates. These characterizations demonstrate the uniformity and the well‐preserved ferroelectricity in these freestanding BFO films.

Zigzag‐wrinkled morphology was found for the freestanding BFO films on blue tape, shown in Figure S3 (Supporting Information). Compared to previous research,^[^ ^27^, ^28^ ^]^ the blue tape is not prestretched in this work. Therefore, the relaxation of the films caused by the release of the compressive strain from the STO substrates is the reason for the occurrence of wrinkle patterns. The thickness plays a vital role in the periodicity of these wrinkles. As we can expect, the thicker is the freestanding BFO films, the larger is the wrinkle period. Specifically, no distinguishable wrinkle can be observed for ≈5‐nm‐thick freestanding BFO films and the wrinkle period is ≈0.79 µm for the ≈20‐nm‐thick ones and ≈1.88 µm for ≈40‐nm‐thick ones. This is due to the largely increased buckling strength.^[^ ^27^ ^]^ We also find that thicker films are brittle and more susceptible to cracking, suggesting their inferior flexibility. This is likely due to the thicker films possessing more internal flaws and domain boundaries, with the fracture generally originating from the nucleation of cracks in proximity to these imperfections.^[^ ^20^ ^]^ As blue tape stretches, the wrinkles in the BFO films gradually spread along the strain direction due to van der Waals interaction between the blue tape and the BFO films. When the maximum strain is reached in both (100) and (010) directions, the wrinkle depth of the two thicker films becomes significantly shallower according to the less contrast of the microscope images, shown in Figure S3 (Supporting Information). As we can see, the ≈5‐nm‐thick films remain relatively flat throughout. Therefore, we use these films for SHG characterization in subsequent tensile strain manipulation experiments.

Anisotropic biaxial strain is employed by a homemade 2D stretching instrument (see Figure S4c in the Supporting Information). Through fixing the blue adhesive plastic film bearing freestanding BFO films on four linear stages and changing the distance of the stages, the nominal strain is firstly applied along (100) direction gradually, up to ≈4% and then added along (010) direction (noted as *x‐*strain and *y‐*strain respectively). Using the marker positioning method, we determine the relationship between the actual strain and nominal strain of the films, as shown in Figure 1f. The tension relation between the tape and the freestanding BFO films is not linear. While the tape stretches up to 4%, the BFO films stretch up to around 1.5%. Up to this point, the films can retain their integrity (see Figure S3 in the Supporting Information for the complete set of stretch) and higher strains increase the likelihood of film cracking, which is consistent with previous studies.^[^ ^21^, ^27^ ^]^

In BFO, the FE polarization directly couples to the G‐type AFM as well as the weak ferromagnetic moment driven by Dzyaloshinskii–Moriya interaction (DMI), stemming from spin‐orbital coupling in asymmetric systems.^[^ ^29^, ^30^, ^31^, ^32^, ^33^, ^34^ ^]^ Thus, the SHG intensity *I_2ω_* and the light‐induced nonlinear polarization ***P*** *_2ω_* in the electric‐dipole approximation have the relationship of *I*_2ω_∝|*P*_2ω_|^2^, where *P*_2ω_ = ε_0_ (χ^(*i*)^ + χ^(*c*)^): *E*_ω_⊗*E*_ω_ with *E_ω_* denoting the incident fundamental light electric field. *χ^(i)^* and *χ^(c)^* are the time‐invariant (*i*‐type) and time‐noninvariant (*c*‐type) SHG tensors, related to the crystallographic (FE) and linear spin‐dependent contributions, respectively.^[^ ^7^, ^25^, ^35^ ^]^ Although less common and often weaker, the electric‐dipole origin of *c*‐type SHG makes it more detectable than higher‐order terms, such as the magnetic‐dipole term and the electric‐quadrupole term. The *c*‐type SHG in BFO is induced by the magnetic structure that simultaneously breaks both space‐inversion and time‐reversal symmetries, leading to asymmetry distortions near the minima in the RA‐SHG patterns. These distortions disappear above the Néel temperature of BFO.^[^ ^7^ ^]^ Therefore, through the strain‐manipulated SHG signals from BFO films, we can identify the variations of FE and AFM orders under the continuous uniaxial and anisotropic biaxial strain. Here, we use a self‐built optical system (see the Experimental Section for details) to perform in situ RA‐SHG measurements. The SHG setup is with a far‐field transmission geometry as sketched in Figure S5a (Supporting Information). The linear dependence of SHG signals with the square of the incident laser power was firstly ensured (shown in Figure S5b in the Supporting Information).

### 2.2 Evolution of FE and AFM Orders of Freestanding BiFeO_3_ Films Probed by SHG

**Figure 2:**
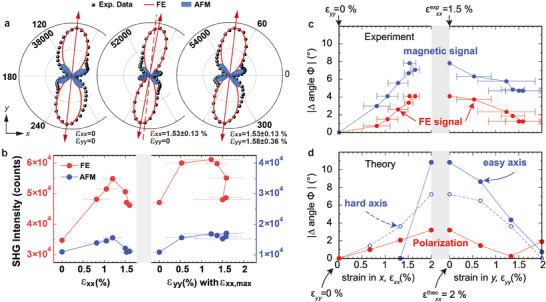
RA‐SHG results of ≈5 nm freestanding BFO film in PAR configuration under anisotropic biaxial strain and theoretical results for FE polarization and AFM structure. a) Evolution of RA‐SHG patterns of the film under different strain. Red arrow indicates the main axis of each pattern. Dashed line is parallel to the left arrow, to better show the rotation. b) Summaries of the extracted peak intensity variations for FE and AFM orders induced SHG components under applied biaxial strains. c) Summaries of the extracted peak position variations for FE and AFM orders induced SHG components under applied biaxial strains. d) Theoretical results of the variations of FE polarization direction and magnetic axis under strain.

By setting the polarization of transmitted harmonic light parallel (PAR) or perpendicular (PER) to the incident fundamental light, then rotating them synchronously, two types of RA‐SHG can be obtained, noted as PAR and PER configuration, respectively. In each configuration, the polarizer and the analyzer rotate synchronously, maintaining the polarization relationship between the incident light and the detected light. **Figure** **2**a exhibit the evolution of RA‐SHG patterns of freestanding BFO films under anisotropic biaxial strain in PAR configuration (see Figure S6 in the Supporting Information for data of all strain values). In RA‐SHG patterns acquired from PAR configuration, the main axis of appearance of the maximum SHG intensity identifies the main direction of symmetry breaking.^[^ ^36^, ^37^, ^38^ ^]^ Here, it corresponds to the net FE polarization direction (projected in *ab*‐plane, red lobes). The RA‐SHG patterns and their normalized contour pattern (see Figure S7a in the Supporting Information) show that the SHG peaks can be distinctly categorized into two types: one possesses the maximum SHG intensity and slight position shift with strain. This shift is induced by the tilt of the FE polarization driven by strain. The other possesses smaller SHG peak intensities with a larger peak position variation. According to previous research,^[^ ^7^, ^25^, ^39^, ^40^, ^41^ ^]^ the asymmetry of the RA‐SHG patterns is a distinct feature for the existence of time‐noninvariant contributions. Further, considering the AFM contribution into the SHG theoretical formula enables a more accurate fit to the experimental data (see Figure 2a and the Experimental Section). It has also been shown previously, this RA‐SHG pattern vanishes at the Néel temperature of BFO (*T_N_* = 618 K).^[^ ^7^ ^]^Therefore, the secondary peaks in the RA‐SHG pattern primarily originate from AFM order.

Figure 2b,c offer clearer illustrations of the variations of SHG peak intensity and position induced by FE and AFM orders, respectively, as strain is applied. Climaxes are found in SHG peak intensity varying with uniaxial strain. When applying *x*‐strain, there are two mechanisms that can affect the in‐plane SHG. One is that the in‐plane strain can drive FE polarization rotating from out‐of‐plane toward in‐plane,^[^ ^27^ ^]^ leading to the increase in ***P*** *_in‐plane_* and the enhancement of SHG signals. The other mechanism is that the stretching of lattice structure along one direction (like *a*) is often accompanied with its contraction in other directions (like *b* or *c*) by strain‐shear coupling,^[^ ^42^, ^43^ ^]^ which leads to the decrease in *y* component of ***P*** *_in‐plane_* and the decrease of SHG signals. The competition between these two mechanisms results in the climax of maximum SHG intensity with uniaxial strain, shown as Figure 2b. It can also explain the subsequent climax when applying *y*‐strain: as the difference between *a* and *b* lattice constants of the freestanding BFO films gradually decreases, the *y* component of ***P*** *_in‐plane_* increases while the *x* component decreases. In addition, the twisting of the oxygen octahedra due to in‐plane elongation would also counteract the enlargement of FE polarization to a certain extent. Ultimately, due to the elongation of the whole crystalline structure induced by biaxial strain, the overall FE polarization and total SHG signal are enhanced. Furthermore, the application of uniaxial strain leads to a gradual narrowing of the SHG lobes, signifying a more focused orientation of the orders within the freestanding BFO films, encompassing both FE and AFM orders.

We observe that the FE pattern gradually tilts to the *x‐*axis with the *x‐*strain increasing, reaching a maximum rotation angle of ≈4°, and then return after the subsequent application of *y‐*strain, indicated by grey and red arrows in Figure 2a. It is noteworthy that the SHG peaks induced by AFM order have experienced an approximate 8° shift, twice that from the FE order, which we interpret as a reflection of the rotation of AFM order in the freestanding BFO films under sequential anisotropic biaxial strain due to larger changes in the direction of the magnetic compared to the FE order under strain.^[^ ^3^, ^44^ ^]^ Further theoretical calculations also reveal the changes in the FE and AFM structures in BFO manipulated by anisotropic biaxial strain, which correspond very well with the observations in SHG measurements, shown as Figure 2c,d. Consequently, the observed evolution of RA‐SHG pattern provides a direct visual representation of the FE polarization's rotation, as well as the variation of magnetic axis, in freestanding BFO films under strain.

### 2.3 Theoretical Description

To understand the effect of anisotropic biaxial strain on FE order in freestanding BFO films, we performed computational simulations using atomistic lattice potentials built with a neural network‐based deep learning method of the DeepMD package.^[^ ^45^, ^46^ ^]^ This model is trained on DFT calculations and simulates the lattice and polarization evolution under varying biaxial strain. Crystal structures were optimized for specific strain values (*ε*_xx_ = 0% to 2%, *ε*_yy_ = 0% to 2%) to determine the resulting polarization rotation (see the Experimental Section and Figure S9 in the Supporting Information for further details).

The theoretical results (red points in Figure 2d) reveal a maximum in‐plane polarization rotation of ≈4° at *ε*_xx_ = 2%, *ε*_yy_ = 0%, in very good agreement with the experimental measurements of Figure 2c. Importantly, the agreement between experiment and theory confirms the stability of in‐plane polarization in BFO films under strain and validates the use of SHG in the PAR setting to identify polarization rotation accurately. By combining continuous strain tuning of freestanding films with theoretical modeling, we can map polarization to SHG signals. This approach could detect subtle polarization variations in applications such as piezoelectric actuators or pyroelectric sensors, offering precise strain‐polarization control for advanced devices.

To understand the evolution of the magnetic SHG signal with strain, we have calculated the magnetocrystalline anisotropy energy (MAE) for the range of strain (see methods for details). The MAE is the energy difference associated with different orientations of the magnetic moments relative to the crystallographic axes, characterizing the preferred direction of magnetization in a material, containing the direction of hard axis/plane and easy axis/plane (preferred magnetization direction). In Figure 2d, the blue points show the change in rotation of the hard axis (circles with dashed lines) and easy axis (blue points with solid line) with strain. The change of the hard axis is approximately double of the change of polarization, where the hard axis is following the polarization direction. The easy axis (note that for low strain, we find an easy plane, perpendicular to the hard axis in agreement with previous investigations^[^ ^34^, ^47^, ^48^, ^49^ ^]^) is perpendicular to the hard axis and its change is even larger than that found for the hard axis. Nevertheless, it qualitatively agrees well with the back‐and‐forth rotation of the magnetic SHG signal in Figure 2c.

Attributing the observed magnetic shifts in BFO to a specific source is challenging due to the intricate nature of its magnetic structure. The interplay between DMI, exchange interaction, and magnetocrystalline anisotropy, which can rotate the easy/hard magnetization axis and induce spin cycloid structures and spin canting, is strongly coupled with structural distortions within BFO.^[^ ^48^, ^50^ ^]^ These coupled effects complicate the identification of the primary driver behind the observed shifts in both experiment and MAE calculation. However, the substantial shift in RA‐SHG observed in response to magnetic order clearly demonstrates the magnetic order's sensitivity to external strain stimuli, especially when the strain is anisotropic in *x* and *y* directions. It induces a clear in‐plane rotation of polarization and changes the magnetic order. This effect is less pronounced with isotropic biaxial strain, where ε_*xx*_ = ε_*yy*_ . This observation opens a new avenue for precisely controlling ferroelectricity and magnetism in freestanding films, where uniaxial or anisotropic strain can be readily achieved and continuously tuned. While anisotropic biaxial strain can be realized in epitaxially strained thin films on orthorhombic substrates like GdScO_3_ and DyScO_3_, it is less flexible and more challenging to control.

### 2.4 RA‐SHG Patterns in Perpendicular Configuration

**Figure 3:**
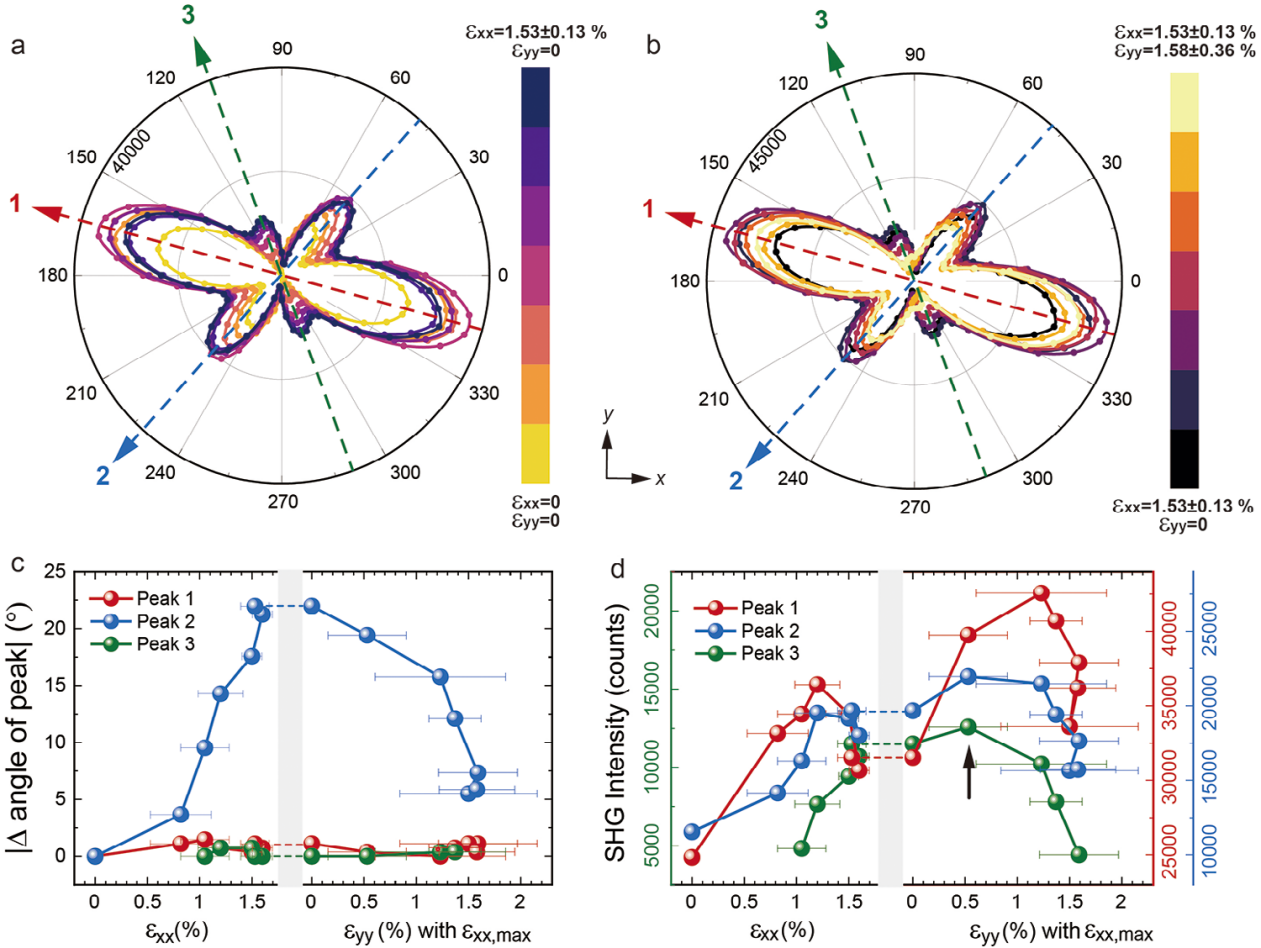
RA‐SHG results of ≈5 nm freestanding BFO film in PER configuration under anisotropic biaxial strain. a, b) Evolution of RA‐SHG patterns under *x*‐strain and *y*‐strain with *ε*_xx,max_, respectively. The arrows note the peak positions. c, d) Summarize of SHG peak positions and intensities noted as peak 1, 2, and 3, respectively. The black arrow indicates where the peak 3 first reached its maximum value.

In addition to the PAR configuration, the RA‐SHG patterns under PER configuration can also provide information related to the structural evolution. As indicated as arrows in **Figure** **3**a,b, there are 3 pairs of peaks in RA‐SHG patterns that changes with strain, noted as peak 1, 2, and 3 for that appearing around 165°/345°, 50°/230°, and 110°/290°, respectively. For peak 1 (red arrow), the intensity is enhanced up to 50.5% with uniaxial strain of about 1.3%. In sharp contrast with these drastic changes in intensity, the peak positions are stable. For peak 2 (blue arrow), despite the same fluctuation trend in intensity under uniaxial strain as peak 1, the position shifts nearly 20° with *x*‐strain increasing and gradually turn back when *y*‐strain was applied, shown in Figure 3c. The changes observed in peaks 1 and 2 are believed to mainly stem from the influence of strain on the FE and AFM orders respectively. Additionally, we consider it possible that substantial alterations in the nonlinear susceptibility tensor may also contribute to the shifts in the RA‐SHG patterns.

An interesting observation is that new peaks appear around 110°/290° under certain strains, namely peak 3 (green arrow). Different from other peaks, the intensity of the new peaks continues to increase with the increasing of lattice anisotropy even when the *y*‐strain is applied, as illustrated in Figure 3d. To understand the origin of Peak 3, we consider the following mechanisms. Given that the RA‐SHG under PAR configuration evolve gradually, it can be ruled out the strain‐induced phase transitions in BFO which usually cause drastic changes in the SHG patterns due to different forms of the nonlinear susceptibilities at different point groups. Mixing phases and multiple ferroelectric domains in BFO could also induce the coupling in its SHG patterns.^[^ ^51^, ^52^ ^]^ However, according to previous research, uniaxial strain is more likely to unify the multiple domains along the strain applied direction, thus reducing the anisotropy. One possible mechanism is the spatial relative shift between the FE and AFM orders. As indicated by the black arrow in Figure 3d, peak 3 reaches its first maximum value under *ε_xx,max_* and *ε_yy_* = 0.53±0.0.37%, near which the difference between the variations of FE and AFM order is the greatest, depending on the theoretical calculation results shown in Figure 2c. In addition, the AFM structure evolution in BFO induced by strain application could also contribute, for example the change from cycloid I to cycloid II (shown as Figure S9 in the Supporting Information), thus resulting in the appearance of SHG peaks at new angles. However, transition between different cycloids occurs at very low tensile strain, about 0.05%.^[^ ^39^ ^]^ Considering the substantial strain applied, a more likely scenario is a transition from cycloidal to (pseudo) collinear AFM order. The mechanism of AFM‐induced SHG has been discussed in several references.^[^ ^7^, ^25^, ^53^, ^54^, ^55^ ^]^ In ref. [49], we have discussed the impact of epitaxial strain on the magnetic ground state.^[^ ^49^ ^]^ With tensile strain, both exchange and DMI decrease linearly and the magnetic ground state results from the sensitive interplay of these interactions and the anisotropy. For BFO, the energy differences between the different cycloids and the G‐Type AFM state are extremely small, hence a change in the magnetic structure is plausible. Our research indicates that apart from the inversion symmetry breaking of AFM order within the crystal lattice due to FE polarization, the spin structure itself, which exhibits a certain independence from the FE order‐induced SHG, also has a significant effect on the AFM‐induced SHG signals. However, whether the strain induced reorientations of FE and AFM orders in BFO are accompanied by the oxygen octahedral tilting and whether the tilting itself can cause a measurable SH signal, as well as the detailed investigation on the magnetic ground state with anisotropic biaxial strain, remain to be investigated.

### 2.5 Photoelastic Tensor and SHG Enhancement

**Figure 4:**
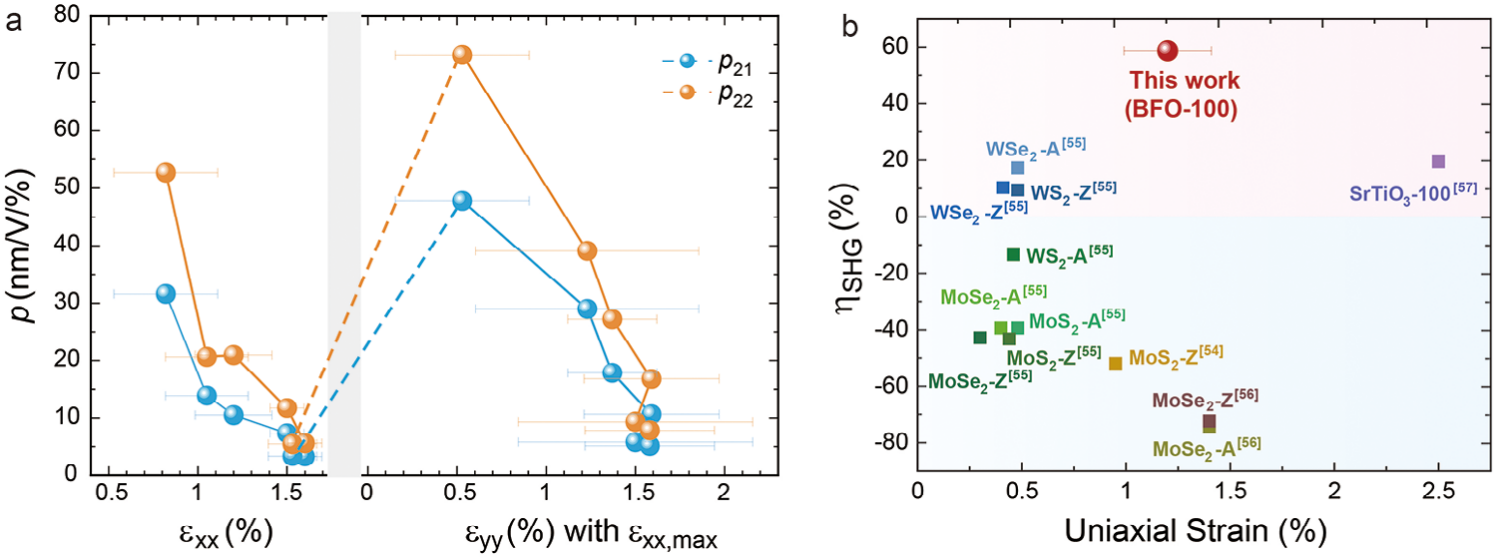
a) The extracted photoelastic tensor elements *p_21_* and *p_22_* depending on the strain. b) Comparison of the SHG enhancement in freestanding BFO films and other reported 2D or freestanding films. −A, –Z, and −100 represent that the strain was applied along armchair, zigzag, and (100) directions, respectively.

In addition to the variations of FE and AFM orders in BFO films under strain illustrated by SHG, we have also found that the BFO thin films exhibit distinctly different macroscopic strain‐manipulated nonlinear optical characteristics compared to 2D layered materials. We introduce the photoelastic tensor *p_jiklm_* to describe the regulation of effective second harmonic susceptibility $\chi_{eff,lab}^{\left(2\right)}$, which translates the strain tensor *u_lm_* into a nonlinear susceptibility contribution, with the form of $p_{ijklm}=\partial \chi_{eff,ijk}^{\left(2,0\right)}/\partial u_{lm}$.^[^ ^56^, ^57^ ^]^ Thus, the tensor of $\chi_{eff,lab}^{\left(2\right)}$ can be written as $\chi_{eff}^{\left(2\right)}=\chi_{eff,0}^{\left(2\right)}+p_{ijklm}u_{lm}$, where *u_lm_* is the strain tensor and $\chi_{eff,0}^{\left(2\right)}$ is the effective nonlinear susceptibility of BFO films in laboratory coordinate system without strain. By analyzing the SHG intensity at some specific light polarization state, we can obtain the corresponding *p_21_* and *p_22_* varying with strain, as shown in **Figure** **4**a. The *p_21_* and *p_22_* are all positive and decrease as the strain increases. This is different to 2D transition‐metal dichalcogenides (TMDCs) materials, in which the *p_ijklm_* is usually negative and remains very stable over a certain range of strain.^[^ ^56^, ^57^, ^58^ ^]^ We think that this is due to the nature of the freestanding perovskite films, the third direction of space can compensate for the control in the *ab*‐plane. The stronger photoelastic property of this freestanding perovskite films under lower strain highlights their advantages in the application of light detection for weak deformation, such as enhanced sensitivity and more tunable optoelectronic properties.

It is also worth noting that a significant enhancement of SHG intensity up to 58.7% is detected under the strain of ≈1.2%, which is the highest enhancement in the SHG signal reported to the best of our knowledge in 2D or freestanding films induced by the direct strain applied through a flexible substrate,^[^ ^59^ ^]^ shown in Figure 4b. Even the tensile strain is about ≈0.4%, the intensity of SHG signal from the freestanding BFO films is enhanced by ≈30%. Moreover, conversely to the strain‐induced attenuation of SHG intensity commonly reported for 2D materials such as TMDCs, our study notes an enhancement of the entire RA‐SHG pattern, not confined to a particular polarization. The variance in SHG modulation by strain between freestanding perovskite oxide films and TMDCs should stem from their fundamental symmetry breaking structures. In ferroelectric perovskite oxides, strain enhances electric dipole moments by elongating the crystal lattice and thus SHG. However, in TMDCs, where atomic arrangement dictates the symmetry breaking, strain can distort the original arrangement, diminishing the SHG signal. The outstanding SHG enhancement in strained freestanding perovskite films positions them as promising candidates for advanced flexible and integrated nonlinear optical devices.

The distinct variation in RA‐SHG patterns under anisotropic biaxial strain is not specific to ≈5 nm freestanding BFO films. For the thicker freestanding BFO films with the thickness of ≈20 nm and ≈40 nm, similar results were observed, as shown in the Supporting Information (Figures S11 and S12, Supporting Information). The initial RA‐SHG patterns are different for each freestanding BFO films, because of their inconsistent net polarization directions. The SHG response to anisotropic biaxial strain is consistent across various film thicknesses, suggesting that the conclusions we have drawn reflect the intrinsic physical property of freestanding BFO films.

The limitation of generalizing the strong spin‐lattice coupling in BFO to other ferroic materials is that the strain response might be different. Because BFO is also a ferroelastic material with excellent flexibility,^[^ ^60^ ^]^ it can be well stretched and characterized in situ (reversible behavior has been observed at strains up to 2.5%). Freestanding ferroic oxide films remain the most promising system for in situ stretching and characterization, as the large strains of freestanding La_0.7_Ca_0.3_MnO_3_ films reported so far is as high as 8%,^[^ ^61^ ^]^ which is a worthy goal for other oxide systems, and freestanding BaTiO_3_ films with super‐elasticity.^[^ ^62^ ^]^ Recent work on in situ stretching and characterization of freestanding PbTiO_3_ films also demonstrates the potential of this approach to be extended to other material systems.^[^ ^62^ ^]^ The biggest challenge probably lies in how to improve the flexibility of the system under study. In the future, perhaps we can reduce the thickness of the freestanding film to maximize the flexibility of the sample and effectively apply stress.^[^ ^21^ ^]^

## 3 Conclusions

We report a systematic study on the evolution of FE and AFM orders in freestanding BFO films under continuous anisotropic biaxial strain using SHG technology and first‐principles calculations. The reversible rotation of the RA‐SHG patterns suggests that the ***P_in‐plane_*** in BFO can be manipulated by the anisotropic strain applied along the (100) and (010) directions, though constrained tilting within ≈4°. In contrast, the strain has a more considerable influence on the AFM order, including an ≈8° rotation in AFM order induced component of RA‐SHG and a reorientation of the spin structure. Our study marks the first observation of the appearance and disappearance of new SHG peaks in BFO films under continuous anisotropic biaxial strain, which can be explained by the spin structure alterations influencing the AFM‐induced SHG signal.

The enhanced SHG signal under strain highlights the potential of freestanding BFO films for applications in 2D nonlinear optical devices, where strain can be used to dynamically control optical properties. Additionally, the nonlinear variation of the photoelastic coefficient under strain suggests that these films are promising candidates for flexible stress sensors with high sensitivity and tunable optoelectronic properties. The ability to manipulate both FE and AFM orders through strain engineering paves the way for the development of multifunctional devices that integrate sensing, actuation, and optical switching into a single material platform. Our research demonstrates that SHG technology is a powerful method for in‐situ and real‐time studies of strain‐manipulated multiferroic 2D materials, opening new avenues for exploring their potential in advanced device applications.

## 4 Experimental Section

### Samples Preparation

Both SAO sacrificial layer and BFO films were deposited on (001)‐oriented STO substrates by Laser‐Molecular Beam Epitaxial (Laser‐MBE) technology using a XeCl excimer laser with the 308‐nm wavelength. The SAO layer was grown at 780 °C under an oxygen pressure of 2 Pa with a substrate‐target distance of 7.5 cm, the laser energy density of ≈2.2 J cm^−2^, and the repetition rate of 2Hz. Subsequently, the BFO layer was deposited at 700 °C under an oxygen pressure of 20 Pa with a substrate‐target distance of 7.5 cm, the laser energy density was ≈1.6 J cm^−2^, and the repetition rate of 2 Hz. After the growth, the heterostructures were annealed in situ at the growth condition of BFO layer for 10 min to maintain surface stoichiometry and then were cooled down to room temperature with a rate of 25 °C min^−1^. A 10 cm × 10 cm × 0.1 mm blue adhesive plastic film was covered on the surface of the BFO/SAO/STO heterostructures. They were then immersed in de‐ionized water at room temperature for about 30–60 min until the SAO sacrificial layer was completely dissolved and the BFO films were separated from the STO substrate. After that, the blue adhesive plastic film together with the freestanding BFO films were dried with N_2_ gas for several seconds.

### Structural and Basic Physical Property Characterization

X‐ray diffraction (XRD), X‐ray reflectivity (XRR), and high‐solution XRD reciprocal space mapping (RSM) techniques were employed to analyze the structures of these BFO films, using a Panalytical X'Pert3 MRD diffractometer with Cu‐Kα1 (1.54056 Å) radiation equipped with a 3D pixel detector. The ferroelectric nature of the freestanding BFO films on Nb‐doped STO conducting substrates was characterized by piezoresponse force microscopy (PFM) performed on a commercial atomic force microscope (AFM, Asylum Research MFP‐3D). The PFM images were collected and recorded using a Ti/Ir‐coated Si cantilever (Olympus Electrilever) with a nominal ≈2 N m^−1^ spring constant and a free air resonance frequency of ≈75 kHz.

### Second Harmonic Generation (SHG) Measurements

The SHG measurements were carried out on a self‐built typical transmissive setup. The fundamental light at 800 nm with 80 MHz repetition rate and 35 fs pulse duration was generated from a Ti:Sapphire oscillator (Spectra Physics) and attenuated to 10 mW before being focused on samples. Under the laboratory coordinate chosen in Figure S5a (Supporting Information), the light was propagating along −*z* direction. The λ/2 waveplate and analyzer loaded on the rotating motors were placed in the incident and transmitted light path respectively, controlled by a computer to rotate the polarization directions of the incident and transmitted light simultaneously and acquire the rotational anisotropy SHG patterns (Note: this is equivalent to rotating the BFO films while keeping the incident and collected polarization fixed). The 800 and 400 nm filters were mounted immediately before and after the samples to ensure the detected SHG is from the BFO films and we confirmed that the blue tape contributes no SHG signal. The SH signal generated from the freestanding BFO films was collected into a monochromator and detected by a photomultiplier tube (PMT). Subsequently, the signal was amplified by a preamplifier and measured by a photon counter to obtain the corresponding SHG intensity.

### SHG Susceptibility

The *T‐*like structure of the ≈5‐nm‐thick freestanding BFO films belongs to the point group of *m*. Considering that the crystal frame does not coincide with the laboratory coordinate system, it is necessary to perform a coordinate transformation. The pseudo‐cubic coordinate system (schematic diagram in Figure S13 in the Supporting Information) is utilized to assist in the coordinate transformation. The transformation matrix from the crystal coordinate system to the laboratory coordinate system ***T*** is in form of

(1) $$T=T^{\prime }T^{\prime \prime }$$

where ***T*″** is the transformation matrix from the crystal coordinate system to the pseudo‐cubic coordinate system and ***T*** ′ is that from pseudo‐cubic coordinate system to the laboratory coordinate system. Thus, the element of effective second order nonlinear susceptibility tensor $\chi_{eff,lab}^{\left(2\right)}$ in laboratory coordinate system is in the form of

(2) $$\chi_{eff,pqr}^{\left(2\right)}=\sum_{i=1}^{3}\sum_{j=1}^{3}\sum_{k=1}^{3}T_{pi}T_{qj}T_{rk}\chi_{ijk}^{\left(2\right)}$$

In the result, $\chi_{eff,lab}^{\left(2\right)}$ is a third‐order tensor with all the elements nonzero, which can also be expressed in the 3×6 matrix format. By submitting it into the SHG intensity expression, the relationship between the sum of the detected frequency‐doubled photon counts and the excitation fundamental light polarization angle can be written as

(3) $$\begin{array}{ccc} S_{∥}^{\left(2\omega \right)} & \propto & [{\chi_{11}}^{\prime }\cos^{3}\alpha +\left(2{\chi_{16}}^{\prime }+{\chi_{21}}^{\prime }\right)\cos^{2}\alpha \sin\alpha \\ & & +\left({\chi_{12}}^{\prime }+2{\chi_{26}}^{\prime }\right)\cos\alpha \sin^{2}\alpha +{\chi_{22}}^{\prime }\sin^{3}{\alpha ]}^{2}\\ S_{⊥}^{\left(2\omega \right)} & \propto & [{\chi_{21}}^{\prime }\cos^{3}\alpha +\left(2{\chi_{26}}^{\prime }-{\chi_{11}}^{\prime }\right)\cos^{2}\alpha \sin\alpha \\ & & +\left({\chi_{22}}^{\prime }-2{\chi_{16}}^{\prime }\right)\cos\alpha \sin^{2}\alpha -{\chi_{12}}^{\prime }\sin^{3}{\alpha ]}^{2} \end{array}$$

for parallel and perpendicular configurations respectively, where *α* is the polarization angle of the incident light. As the noncentrosymmetric magnetic order also breaks the spatial‐inversion symmetry, the magnetization‐induced SHG is produced. The magnetic point group in freestanding BFO films is also *m*, so it has the similar relationship to the above.

### First‐Principles and Atomistic Potential Calculations

Our effective atomistic potential of BFO has been trained on a set of density functional theory (DFT) data using the DeepMD package.^[^ ^44^, ^45^ ^]^ The DFT training set contains about 4500 configurations, sampling a broad range of atomic displacements and strains.^[^ ^63^ ^]^ All these DFT calculations were carried out with ABINIT^[^ ^64^, ^65^, ^66^, ^67^ ^]^ using a LDA+U functional with a Hubbard *U* value of *U*(Fe)  =  4 eV. The DFT dataset encompassed 2 × 2 × 2 pseudocubic supercells of BFO. The model has been carefully validated and appears appropriate to describe accurately a broad range of BFO properties.^[^ ^48^ ^]^ To simulate in‐plane strain effects, we relaxed the 2 × 2 × 2 pseudo‐cubic BFO structures by fixing the lattice constants *a* and *b* while allowing lattice constant *c* to vary freely. While doing the structural optimizations, we also fixed in plane lattice angle (angle between *a* and *b*) to 90°, other two angles between *c* and *a/b* were relaxed. Structural optimizations were carried out using LAMMPS software.^[^ ^68^ ^]^ The macroscopic polarization was estimated using the expression: $\vec{p}=\frac{1}{\Omega }\sum_{i}Z_{i}^{*}({\vec{r}}_{i}-{\vec{r}}_{i}^{ref})$ where Ω is the volume of the simulation cell, $Z_{i}^{*}$ denotes the effective born charge of atom I derived from the cubic phase, (see Table S1 in Supporting Information) ${\vec{r}}_{i}$ and ${\vec{r}}_{i}^{ref}$ represent the atomic positions in the deformed and reference structures, respectively.

For the calculations of the magnetocrystalline anisotropy energy, we have used the structures above and assumed a collinear G‐type AFM state. Using the full potential linearized augmented planewave method^[^ ^69^, ^70^ ^]^ as implemented in the code FLEUR,^[^ ^71^ ^]^ we calculated the energies of different magnetization directions applying second quantization and the force theorem.^[^ ^72^ ^]^ We fitted the energy landscape to the anisotropy energy *E*  =   −*m^T^Km* with *K* corresponding to the magnetocrystalline anisotropy tensor and *m^T^*,*m*, the (transposed) vector of magnetization directions. From that, we have determined the direction of easy and hard axis for every value of strain. Note that for small strain in the *x* direction of Figure 2c, we find an easy plane of magnetization perpendicular to the polarization direction, in agreement with previous studies. Hence, the change in angle of the easy axis cannot be drawn easily in that region. We used computational parameters with LDA+U^[^ ^73^ ^]^ of a Hubbard *U*(Fe)  =  4 eV and *J* (Fe) =  0.4 eV, a planewave cutoff of *k_max_* =  4.6 a.u.^−1^ and 810 k‐points in the full Brillouin zone of the p1 symmetric structural phases of BFO.

## Conflict of Interest

The authors declare no conflict of interest.

## Author Contributions

J.W. and S.X. contributed equally to this work. J.W., K.C., and K.J. conceived and supervised the research project. S.X. prepared the epitaxial heterostructures and biaxial strain applying device. J.W. and S.W. prepared the freestanding films and executed the SHG measurements. J.W., S.X., and Q.M. constructed the theoretical fitting of SHG experimental data and the analysis of optical CCD images. S.M., S.B., X.H., and P.G. performed the first principles based calculations. S.H., S.X., and, E.G., carried out the XRD and PFM characterizations. J.W., S.X., Q.M., P.L. drew the illustration figures. All the authors discussed the results and commented on the manuscript.

## Supporting information



Supporting Information

## Data Availability

The data that support the findings of this study are available in the supplementary material of this article.
