# Supplementary material for: Manipulation of Ferroic Orders via Continuous Biaxial Strain Engineering in Multiferroic Bismuth Ferrite
Source: Adv Sci (Weinh). 2025 Mar 26;12(19):2417165. doi: 10.1002/advs.202417165 (PMC12097119; doi:10.1002/advs.202417165)
Supplement: Supplementary file 1 — Supporting Information [file ADVS-12-2417165-s001.docx]

***Supporting Information***

**Manipulation of Ferroic Orders via Continuous Biaxial Strain Engineering in Multiferroic Bismuth Ferrite**

*Jiesu Wang^1,†,*^, Shuai Xu^2,†^, Sebastian Meyer^4^, Shiyao Wu^1^, Subhadeep Bandyopadhyay^5^, Xu He^5^, Qiyuan Miao^1,6^, Sisi Huang^2^, Pengzhan Li^2^, Kun Zhao^6^, Er-Jia Guo^2,3^, Chen Ge^2,3^, Bertrand Dupé^4^ Philippe Ghosez^5^, Kai Chang^1,*^, and Kuijuan Jin^2,3,*^*

^1^Beijing Academy of Quantum Information Sciences, 100193 Beijing, China

^2^Beijing National Laboratory for Condensed Matter Physics, Institute of Physics, Chinese Academy of Sciences, 100190 Beijing, China

^3^University of Chinese Academy of Sciences, 100049 Beijing, China

^4^TOM research group, Q-MAT research unit, Université de Liège, Liège B-4000, Belgium

^5^Theoretical Materials Physics, Q-MAT research unit, Université de Liège, Liège B-4000, Belgium

^6^Institute of Ultrafast Optical Physics, Department of Applied Physics and MIIT Key Laboratory of Semiconductor Microstructure and Quantum Sensing, Nanjing University of Science and Technology, 210094 Nanjing, China

^†^These authors contributed equally: Jiesu Wang, Shuai Xu.

*Corresponding Authors

E-mail: wangjs@baqis.ac.cn (J. W.)

E-mail: changkai@baqis.ac.cn (K. C.)

E-mail: kjjin@iphy.ac.cn (K. J.)


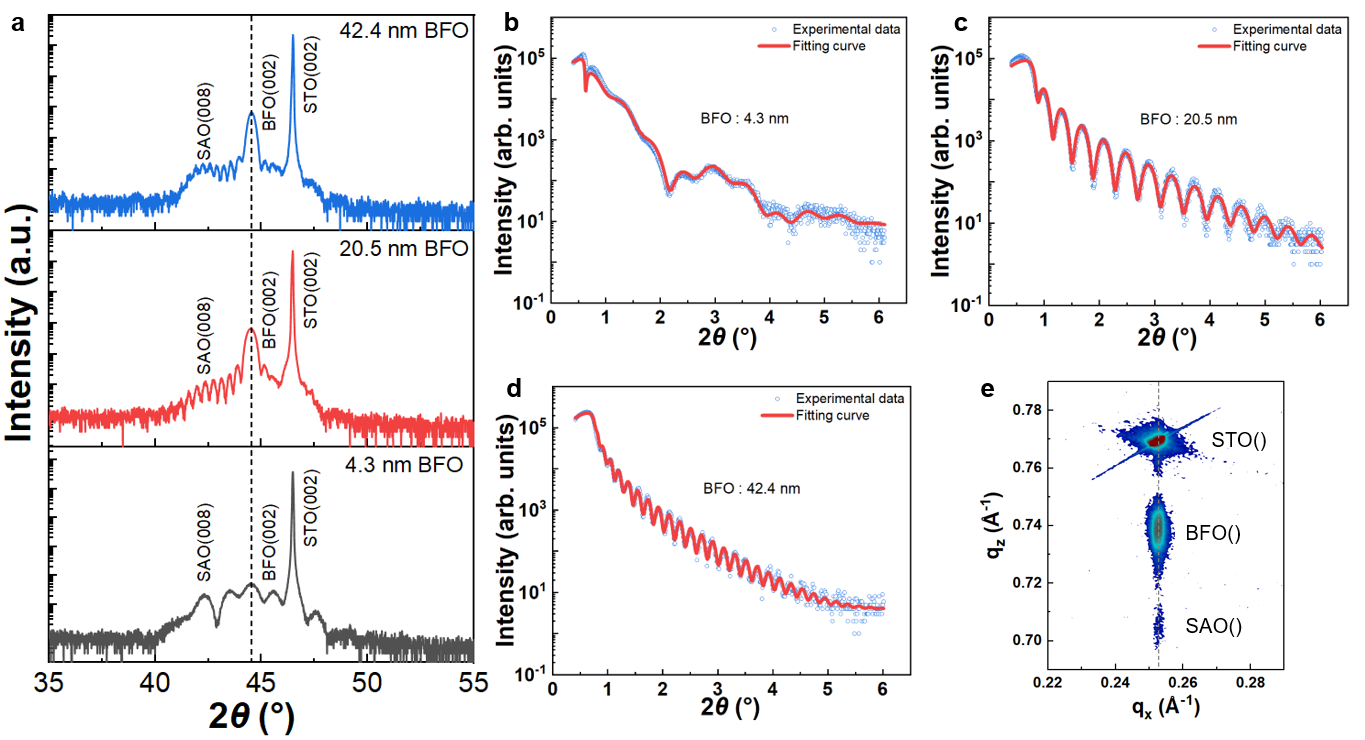


**Figure S1.** XRR and XRD results. a) X-ray diffraction curves of BFO/SAO/STO heterostructures with BFO thicknesses of 4.2 nm, 20.5 nm, and 42.4 nm, respectively determined by the X-ray reflectivity measurements shown in (b, c, and d**)**. The dashed line indicates the (002) diffraction peaks of BFO layer. Because of the existence of SAO buffer layer, these peaks remain consistent throughout the thicknesses variation. d) reciprocal space mapping (RSM) results of the as-grown ~20-nm-thick BFO/SAO/STO heterostructure, proving the well epitaxial growth of both BFO and SAO layers.


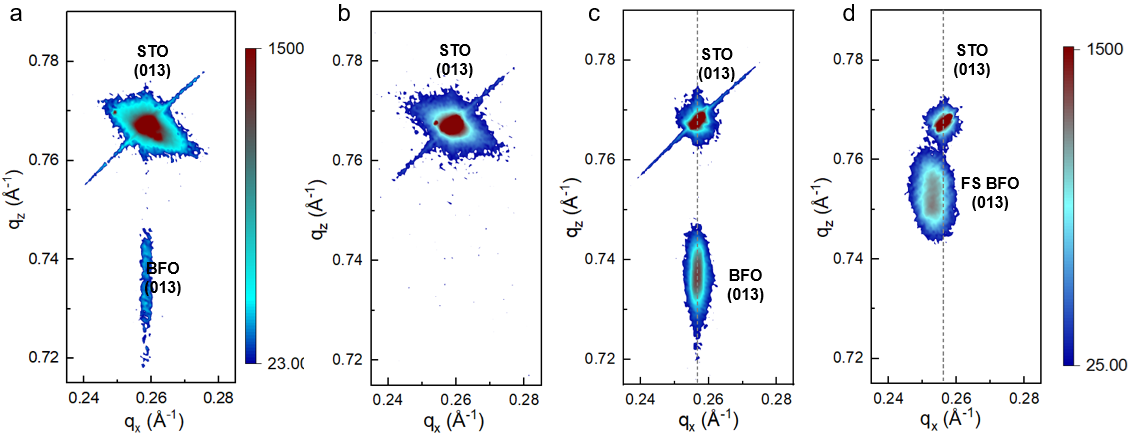


**Figure S2.** a,b) RSM results of ~5-nm-thick epitaxial and freestanding BFO films around (013) peak, respectively. c,d) RSM results of ~40-nm-thick epitaxial and freestanding BFO films around (013) peak, respectively. No significant diffraction spots are seen in the mapping image, because the thickness of ~5 nm is too thin to provide the detectable X-ray diffraction signal. The thicker films exhibit obvious diffraction spots. Although the spot area increased, no splitting occurred, indicating the relaxation of the in-plane strain and the consistency of the structure in BFO films. ^[1-4]^


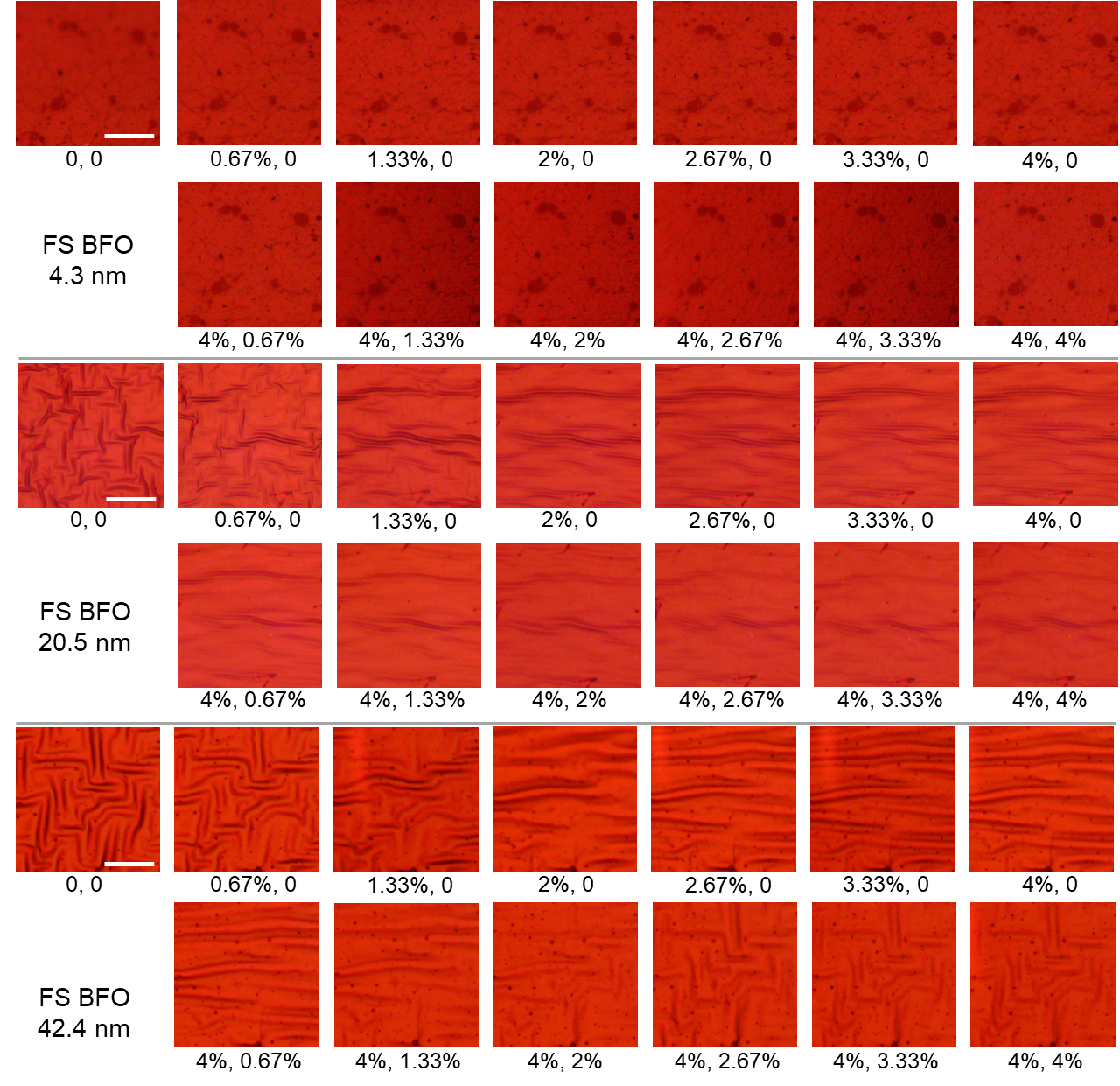


**Figure S3.** In-situ CCD images of freestanding BFO films in SHG light path under anisotropic biaxial strain with the thickness of ~4 nm, ~20 nm, and ~40 nm respectively. Scale bars are 10 μm. The numbers below each image represent corresponding nominal strain along *x-* and *y-* directions.


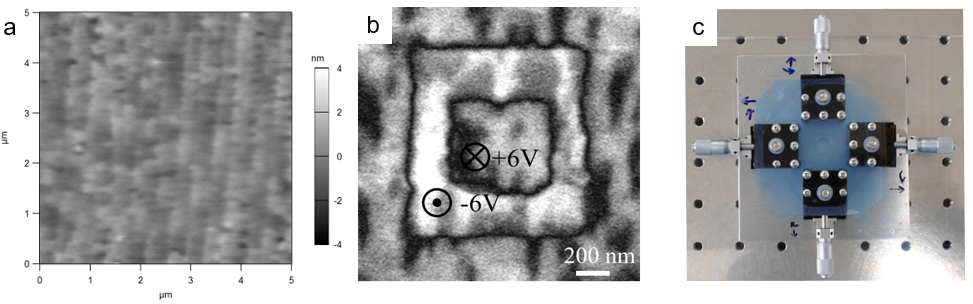


**Figure S4.** a,b) Surface atomic force microscopy (AFM) and out-of-plane PFM amplitude images of ~20-nm-thick freestanding BFO films after poled by DC voltage of ± 6 V, respectively. c) Homemade instrument for applying two-dimensional strain to freestanding films in transmissive light path.


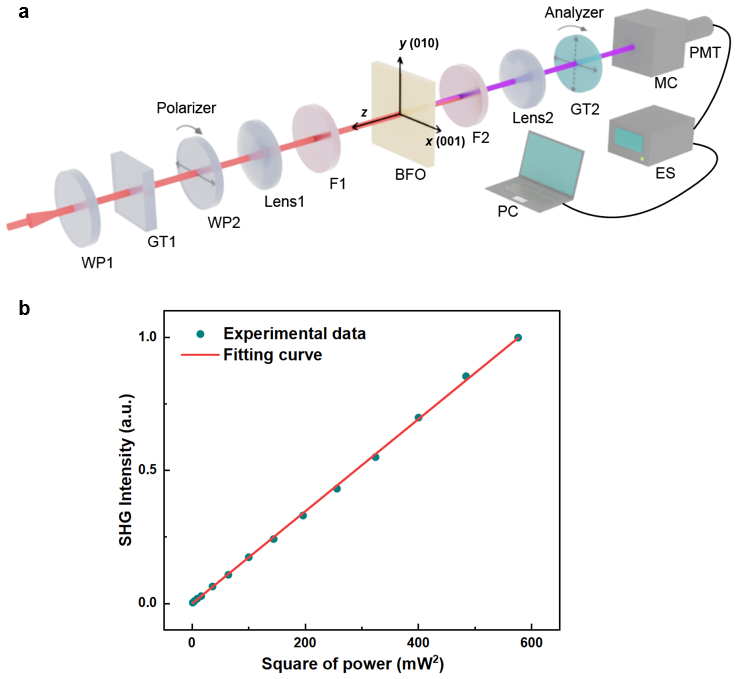


**Figure S5.** a) Schematic diagram of the far-field transmission RA-SHG system. A half-wave plate (WP1) and a Glan-Taylor prism (GT1) are used to adjust the intensity and ensure the horizontal polarization of the incident light. Then, an electrically controlled half-wave plate (WP2) is used to modulate the light polarization and a convex lens (Lens1) is engaged to focus light onto the sample. Filter F1 removes any second harmonic light generated in the polarization optics before the sample. The fundamental frequency and higher-order harmonics in the emitted light are blocked by a 400 nm filter (F2). The generated SH signal is collected by a convex lens (Lens2) and the polarization state is analyzed through an electrically rotated Glan-Taylor prism (GT2). The SHG intensity is integrated by electronic system after passing a monochromator (MC) and photomultiplier tube (PMT). For PAR configuration, the initial polarizations of incident and transmissive light are parallel (as indicated by the solid arrow in WP2 and GT2). For PER configuration, the initial polarizations of incident and transmissive light are perpendicular (as indicated by the solid arrow in WP2 and dashed arrow in GT2). b) Linear relationship between the optical intensity at 400 nm and the square of incident light power, confirming the SHG responses. For other samples, they exhibit the similar properties.


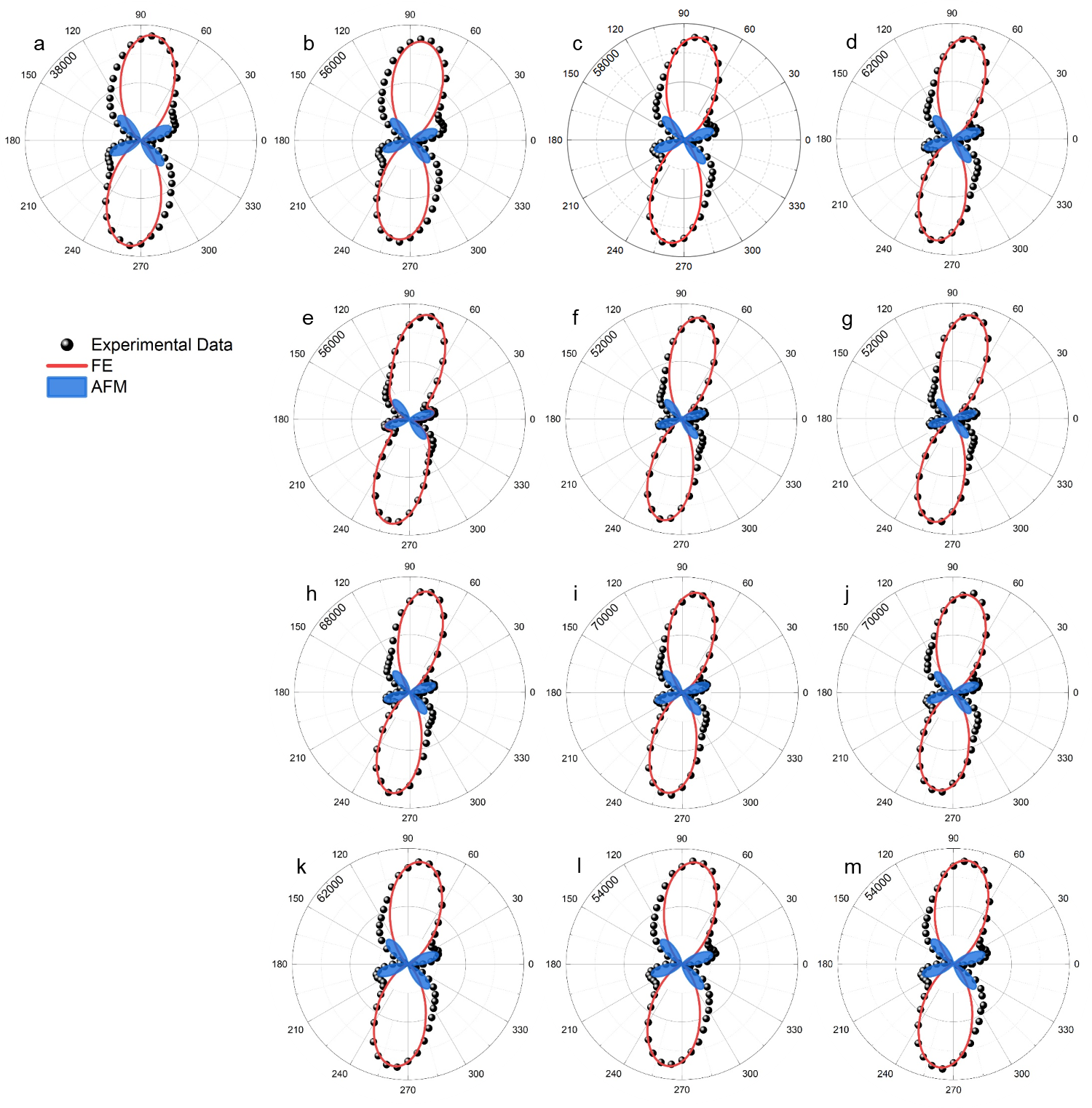


**Figure S6. The results of extracting the SHG signals induced by FE and AFM mechanisms from the RA-SHG patterns of ~5 nm freestanding BFO films under different strains in PAR configuration.** a-g) correspond to the uniaxial nominal *x*-strain of 0, 0.67%, 1.33%, 2%, 2.67%, 3.33% and 4% with no *y*-strain applied, respectively. h-m) correspond to the nominal *y*-strain of 0, 0.67%, 1.33%, 2%, 2.67%, 3.33% and 4% with nominal *x*-strain of 4%, respectively.


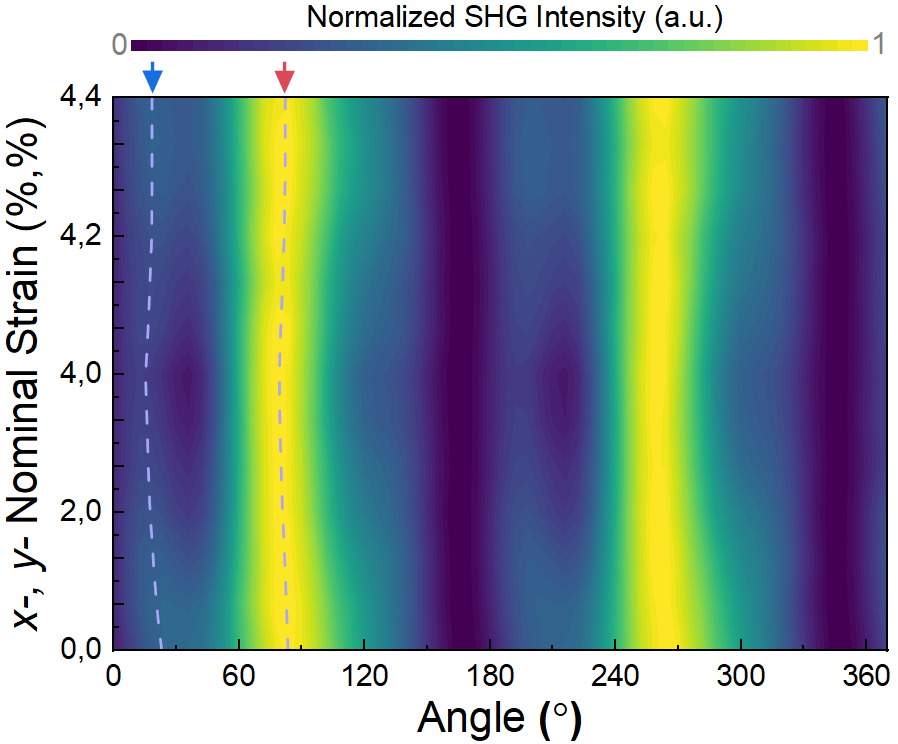


**Figure S7. Normalized contour patterns of RA-SHG for the ~5-nm-thick freestanding BFO films under** **anisotropic biaxial strain in PAR configuration.** Dashed lines indicate the SHG peak positions. Red and blue arrows show the SHG peaks induced by FE and AFM orders respectively.


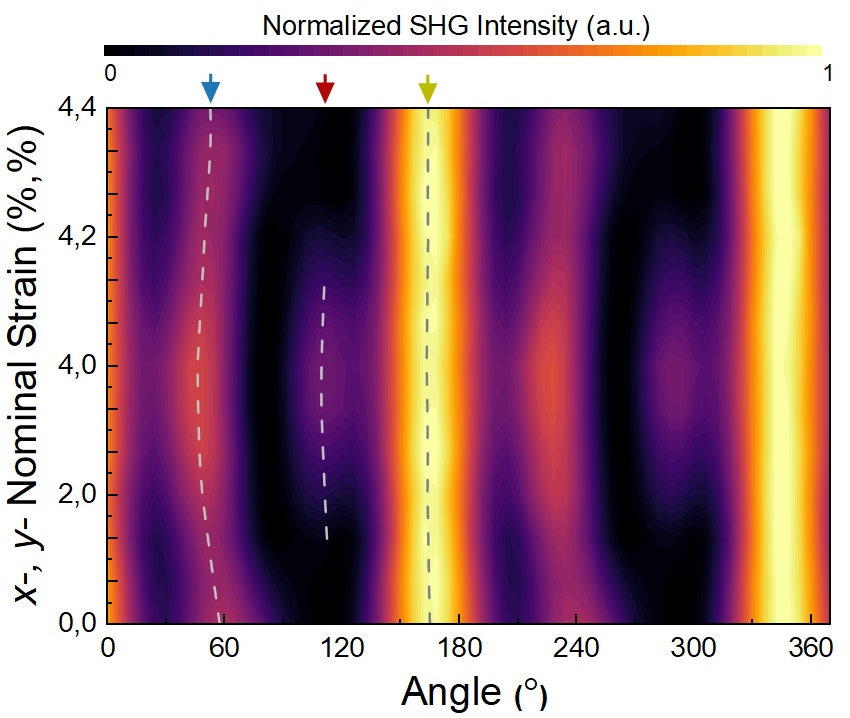


**Figure S8. Normalized contour patterns of RA-SHG for the ~5-nm-thick freestanding BFO films under anisotropic biaxial strain in PER configuration.** Dashed lines indicate the SHG peak positions. The yellow, blue, and red arrows are corresponding to the peak 1, 2, and 3 respectively in the manuscript.


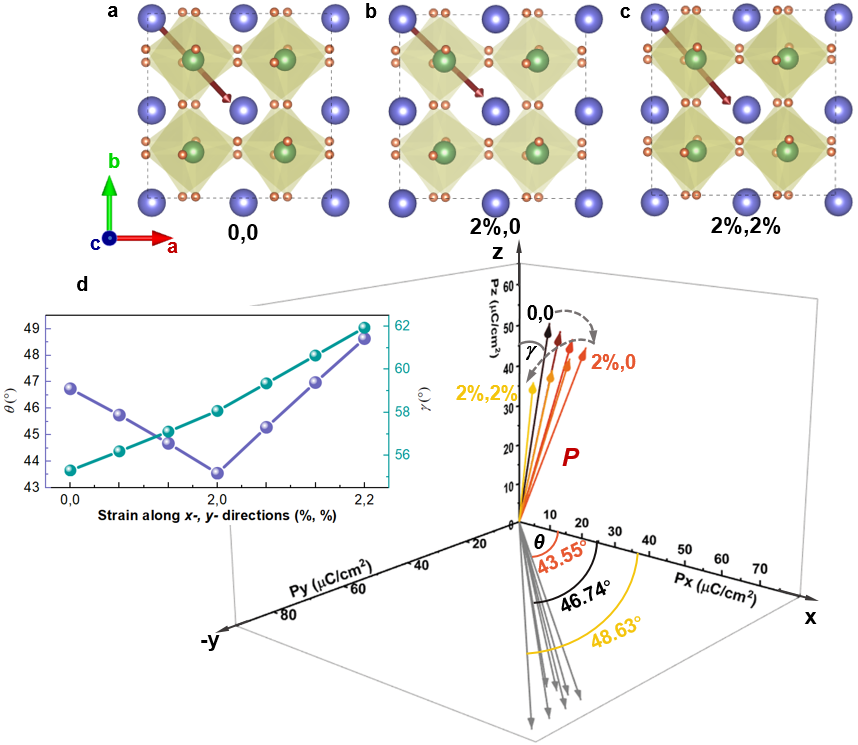


**Figure S9. Theoretical results for the evolution of ferroelectric polarization in BFO films under anisotropic biaxial strain up to 2%.** a,b,c) Top views of the atomic structures and ferroelectric polarizations of BFO under the x-/y- axis strain of 0/0, 2%/0, and 2%/2%, respectively. The red arrows merely indicate the directions of polarizations but not the strength. d) The sketch of ferroelectric polarization gradually varying with anisotropic biaxial strain in the pseudocubic coordinate system. The inset shows the variation of the angle between ***P_in-plane_*** and *x*-axis depending on the anisotropic biaxial strain.


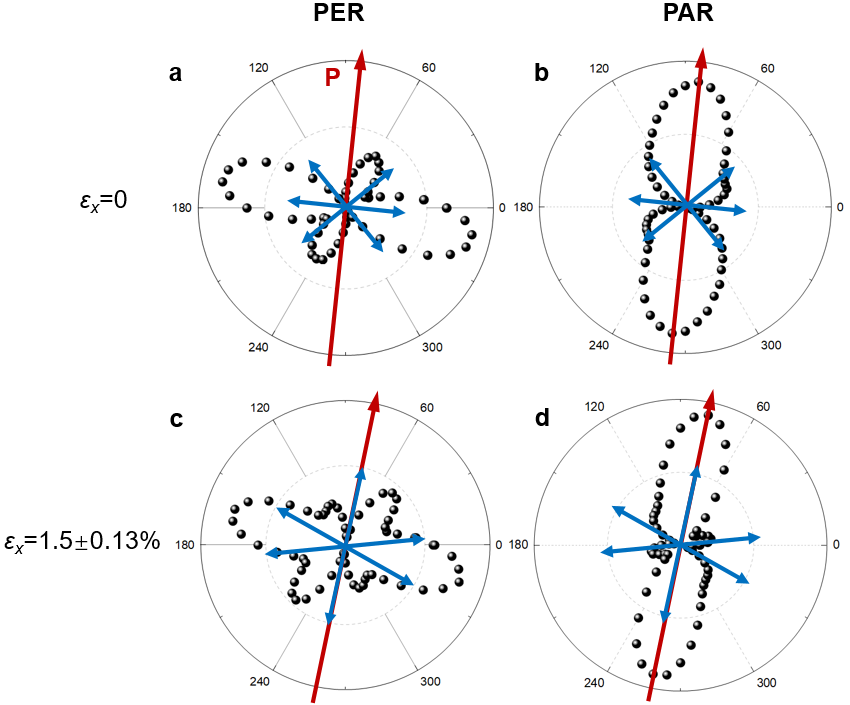


**Figure S10. Schematic diagram of the possible correspondence between FE and AFM orders and RA-SHG patterns in freestanding BFO films if a transition from cycloid Ⅰ to cycloid Ⅱ occurs under uniaxial strain.** Red and blue arrows represent for the FE polarization direction and corresponding three possible propagation directions ***k*** for AFM cycloid order projected in plane, respectively. ^[5]^


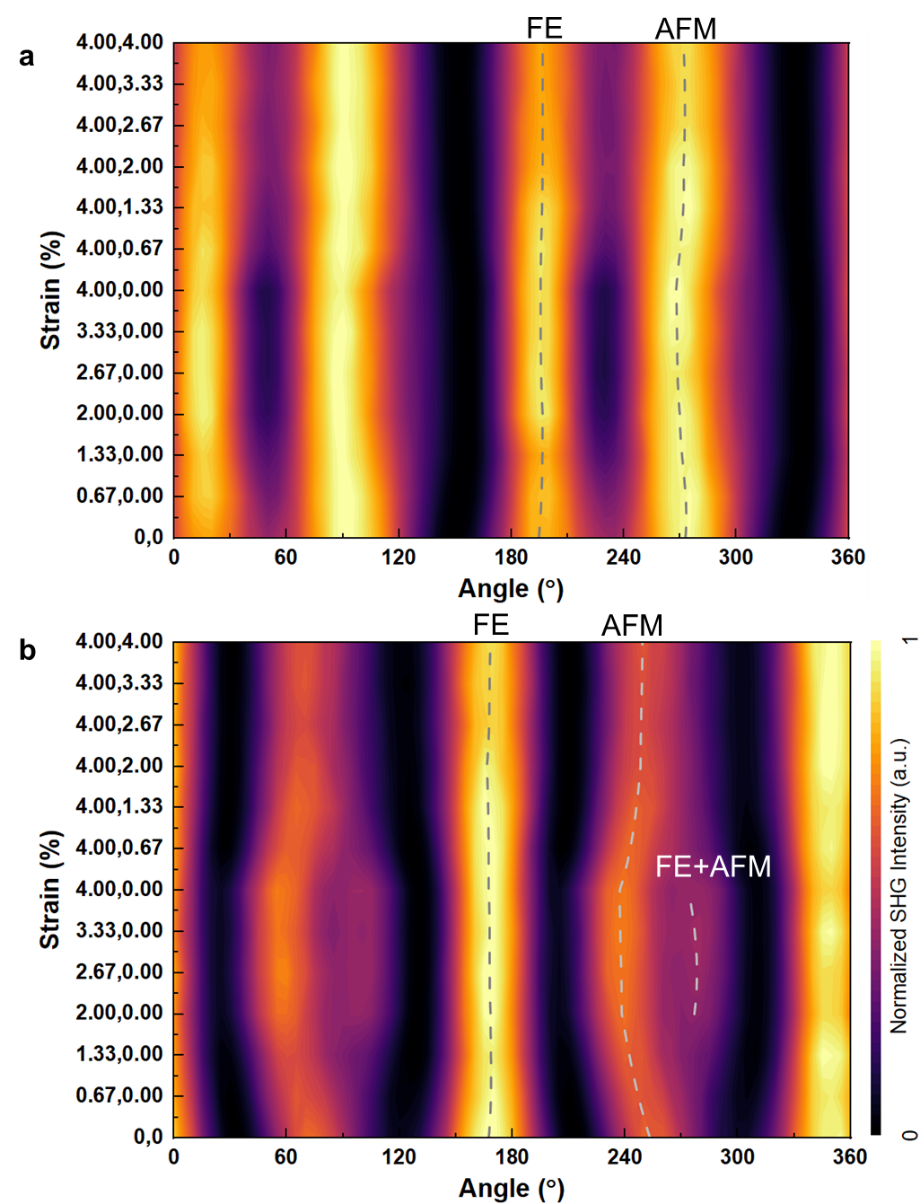


**Figure S11.** Normalized contour patterns of RA-SHG for the thicker freestanding BFO films with the thickness of ~20 nm under anisotropic biaxial nominal strain in a) PAR and b) PER configurations.


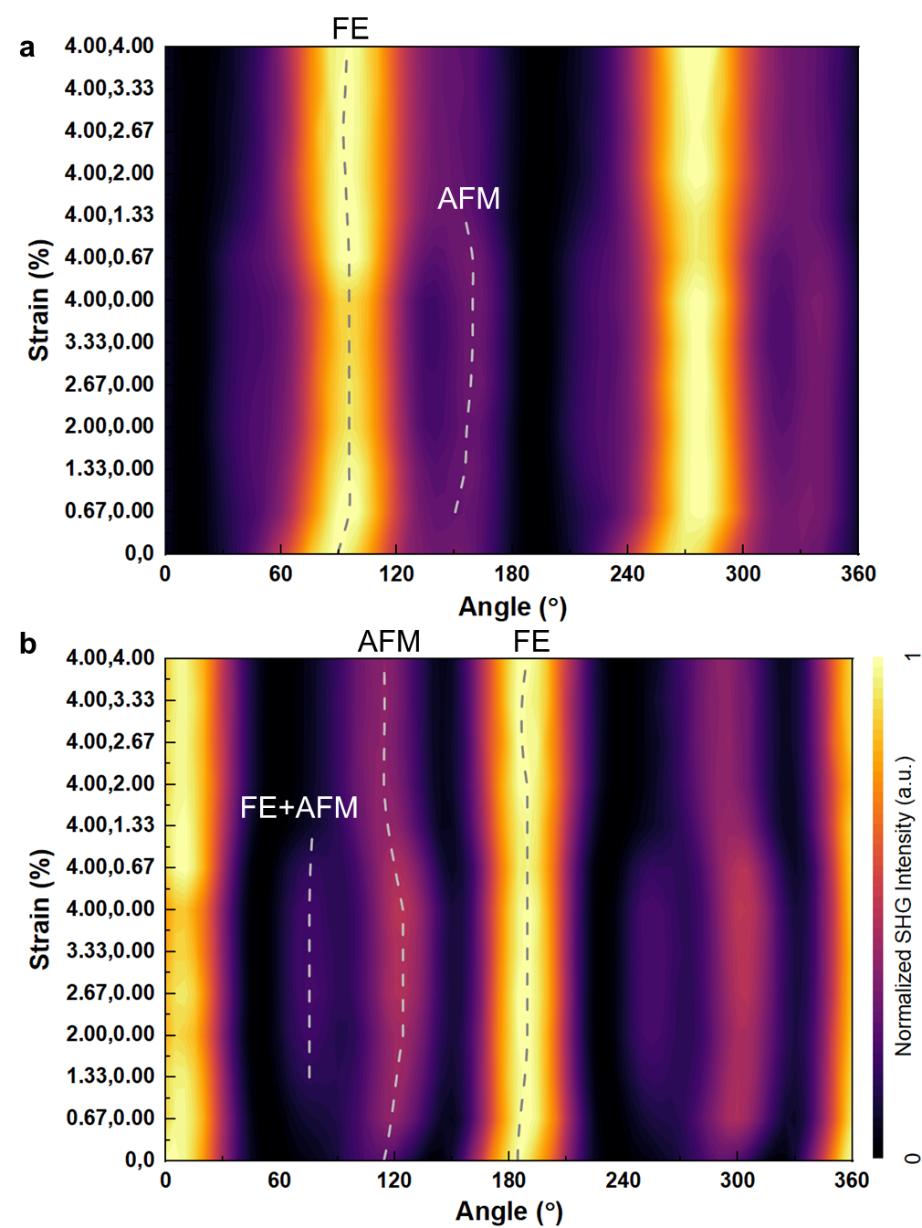


**Figure S12.** Normalized contour patterns of RA-SHG for the thicker freestanding BFO films with the thickness of ~40 nm under anisotropic biaxial nominal strain in a) PAR and b) PER configurations.


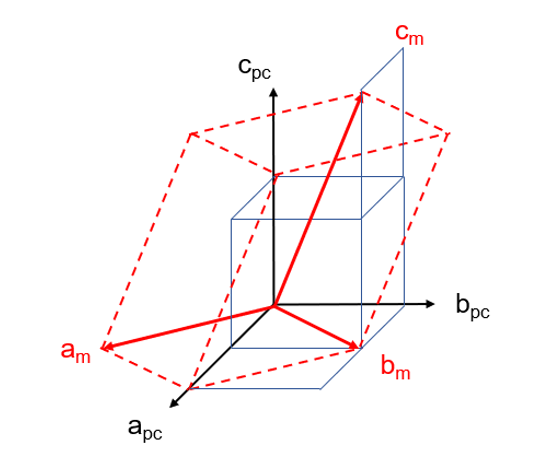


**Figure S13.** Schematic diagram for the correlation between the crystallographic coordinate system (red) and the pseudo-cubic coordinate system (black) of monoclinic BFO structures. In SHG theoretical analysis part, we assume that *a_pc_‖x*-axis and *b_pc_*⊥*y*-axis.

**Table S1:** The born effective charges of Bi, Fe and O in the cubic BFO structure. Only one of the O is included here while the others can be obtained by symmetry operation.

| **Bi** | | |
| --- | --- | --- |
| 6.339 | 0 | 0 |
| 0 | 6.339 | 0 |
| 0 | 0 | 6.339 |
| **Fe** | | |
| 3.947 | 0 | 0 |
| 0 | 3.947 | 0 |
| 0 | 0 | 3.947 |
| **O (O in the z-direction)** | | |
| -3.481 | 0 | 0 |
| 0 | -3.481 | 0 |
| 0 | 0 | -3.324 |

**References**

[1] K. Saito, A. Ulyanenkov, V. Grossmann, H. Ress, L. Bruegemann, H. Ohta, T. Kurosawa, Sadao Ueki, H. Funakubo, *Japanese Journal of Applied Physics Part 1-Regular Papers Brief Communications & Review Papers* **2006**, *45*, 7311.

[2] J. S. Wang, K. J. Jin, H. Z. Guo, J. X. Gu, Q. Wan, X. He, X. L. Li, X. L. Xu, G. Z. Yang, *Scientific Reports* **2016**, *6*, srep38268.

[3] S. Xu, J. S. Wang, P. Chen, K. J. Jin, C. Ma, S. Y. Wu, E. J. Guo, C. Ge, C. Wang, X. L. Xu, H. Yao, J. Y. Wang, D. Xie, X. Wang, K. Chang, X. D. Bai, G. Z. Yang, *Nature Communications* **2023**, *14*, 2274.

[4] C. W. Huang, L. Chen, *Materials* **2014**, *7*, 5403.

[5] A. Haykal, J. Fischer, W. Akhtar, J.-Y. . Chauleau, D. Sando, A. Finco, F. Godel, Y. A. Birkhölzer, C. Carrétéro, N. Jaouen, M. Bibes, M. Viret, S. Fusil, V. Jacques, V. Garcia, *Nature Communications* **2020**, *11*, 1704.
